# The impact of socioeconomic status on survival in stage III colon cancer patients: A retrospective cohort study using the SEER census‐tract dataset

**DOI:** 10.1002/cam4.4099

**Published:** 2021-06-30

**Authors:** Amina Dhahri, Jori Kaplan, Syeda M. H. Naqvi, Naomi C. Brownstein, Shana O. Ntiri, Iman Imanirad, Seth I. Felder, Sean P. Dineen, Julian Sanchez, Sophie Dessureault, Estrella Carballido, Benjamin D. Powers

**Affiliations:** ^1^ Department of Internal Medicine University of Maryland Capital Region Health Largo MD USA; ^2^ Department of Hematology and Medical Oncology Moffitt Cancer Center University of South Florida Tampa FL USA; ^3^ Department of Biostatistics and Bioinformatics Moffitt Cancer Center Tampa FL USA; ^4^ University of Maryland School of Medicine Baltimore MD USA; ^5^ The University of Maryland Greenbaum Comprehensive Cancer Center, University of Maryland Greenbaum Comprehensive Cancer Center Baltimore MD USA; ^6^ Health Outcomes and Behavior Program Moffitt Cancer Center Tampa FL USA

**Keywords:** census tract, SEER, socioeconomic, stage III colon cancer, survival

## Abstract

**Background:**

The impact of socioeconomic status (SES) has been described for screening and accessing treatment for colon cancer. However, little is known about the “downstream” effect in patients who receive guideline‐concordant treatment. This study assessed the impact of SES on cancer‐specific survival (CSS) and overall survival (OS) for stage III colon cancer patients.

**Methods:**

The SEER Census Tract‐Level SES Dataset from 2004 to 2015 was used to identify stage III colon adenocarcinoma patients who received curative‐intent surgery and adjuvant chemotherapy. The predictor variable was census tract SES. SES was analyzed as quintiles. The outcome variables were OR and CSS. Statistical analysis included chi square tests for association, Kaplan–Meier, Cox, Fine and Gray regression for survival analysis.

**Results:**

In total, 27,222 patients met inclusion criteria. Lower SES was associated with younger age, Black or Hispanic race/ethnicity, Medicaid/uninsured, higher T stage, and lower grade tumors. CSS at the 25th percentile was 54 months for the lowest SES quintile and 80 for the highest. Median OS was 113 months for the lowest SES quintile and not reached for highest. The 5‐year CSS rate was 72.4% for the lowest SES quintile compared to 78.9% in the highest (*p* < 0.001). The 5‐year OS rate was 66.5% for the lowest SES quintile and 74.6% in the highest (*p* < 0.001).

**Conclusion:**

This is the first study to evaluate CSS and OS in an incidence‐based cohort of stage III colon cancer patients using a granular, standardized measure of SES. Despite receipt of guideline‐based treatment, SES was associated with disparities in CSS and OS.

## Graphical abstract

This is the first study to evaluate CSS and OS in a national cohort of stage III colon cancer patients using a granular, standardized measure of SES. We showed that despite receipt of guideline‐based treatment, socioeconomic status is associated with disparities in overall and cancer specific survival. These findings suggest opportunities to improve colon cancer outcomes farther down the cancer care continuum.

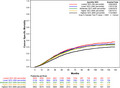

## 1 INTRODUCTION

In the United States, colorectal cancer is the second most common cause of cancer mortality.^1^ Encouragingly, the death rate for colorectal cancer has been in decline since 2000 and the 5‐year survival rate has reached 64.6%.^2^ This has been attributed in large part to greater screening and risk factor modifications; however, improved treatment has also been reported to play a role.^2^ Despite these advances, receipt of guideline‐based treatment for colon cancer remains inequitable as low SES, under and uninsured, and minority patients have been shown to receive less treatment and have worse survival.^3^, ^4^

While much focus has been placed on modifying risk factors and early detection through screening, few studies have evaluated disparities in colon cancer outcomes farther down the care continuum, such as follow‐up and treatment of recurrence.^3^ Stage III colon cancer is diagnosed when disease has spread to the regional lymph nodes and is associated with a higher risk of early recurrence.^4^ To mitigate this risk, adjuvant chemotherapy has been shown to improve disease‐free and overall survival for stage III colon cancer and is the standard of care.^5^, ^6^

While studies from randomized trials have shown that African‐Americans receiving adjuvant therapy for colon cancer have similar outcomes to Caucasian patients, incident and population‐based registries have demonstrated significant socioeconomic and race disparities.^7^, ^8^, ^9^, ^10^ These findings suggest that unequal cancer care may be driving these disparities, including post‐treatment surveillance, and treatment of recurrence.^11^, ^12^ With improvements in lymph node evaluation for localized disease and treatment of peritoneal and liver metastasis, post‐treatment follow‐up and surveillance and referral for treatment of recurrence is increasingly important.^13^, ^14^, ^15^ Nevertheless, data show that roughly 75% of post‐surgical patients do not receive the minimum recommended surveillance.^16^

Therefore, this study used a nationally representative, incidence‐based dataset to assess risk factors associated with CSS and OS in a cohort of stage III colon cancer patients who received standard of care treatment. Specifically, we focused on the role of census‐tract SES as a driver of these outcomes. We hypothesized that despite accessing guideline‐concordant care, the downstream effect of low socioeconomic status would negatively impact survival.

## 2 METHODS

### 2.1 Study cohort and setting

The Surveillance, Epidemiology, and End Results Program (SEER) Census Tract‐Level SES and Rurality Database was used to identify colon adenocarcinoma patients diagnosed from 2004 to 2015 with resected stage III disease who initiated adjuvant therapy. The SEER Census Tract‐Level SES and Rurality Database is a population‐based cancer registry which captures approximately 34% of the United States population and is generally representative of the demographics of the US population.^17^ Because the data were de‐identified, the study was exempt from review by the Moffitt Cancer Center IRB.

**FIGURE 1:**
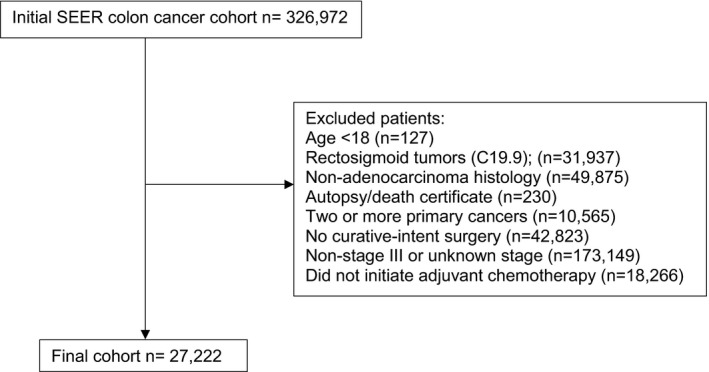
Study population selection criteria

Colon adenocarcinoma cases were selected for analysis (Table S1). C19.9‐Rectosigmoid junction cases were excluded. Race and ethnicity were assessed using the race and Hispanic origin variable. Patients who underwent surgery of the primary site were included (codes 30–80). Stage III patients were included based pathologic staging; patients with metastatic disease or in situ disease were excluded. Patients who initiated chemotherapy were included; cases without initiation of chemotherapy were excluded. A summary of exclusion data is presented in Figure 1.

### 2.2 Predictor variable

The predictor variable was census‐tract level SES. The NCI’s census tract‐level SES index is constructed using factor analysis of seven variables based on data from Yost.^18^ The variables include median household income, median house value, median rent, percent below 150% of poverty line, an education index, percent working class, and percent unemployed. After annual SES scores are generated, the census tracts are categorized quintiles and tertiles with equal populations. For this study, quintiles were used for analysis.

### 2.3 Outcomes variables

The outcome variables included OS and CSS. OS was defined as months from the date of colon cancer diagnosis to the date of death from any cause or was censored at the date of last contact. Cancer‐specific mortality was defined based on the SEER code for cause‐specific death classification as to whether the death was attributable to the primary cancer diagnosis or a cause different from the primary cancer.

### 2.4 Covariates

Covariates were included as potential confounders. Demographic factors including age, sex, race, ethnicity, insurance status, and urban/rural residence were included. Race is categorized in SEER through medical record abstraction. Ethnicity was defined as Hispanic based on SEER assignment through self‐report of Spanish origin in the medical record or by a computer algorithm that searches surnames and maiden names to determine Spanish origin. Individuals of Spanish origin were categorized as Hispanic, regardless of racial background. Due to the change in availability of insurance status around 2007, insurance status was classified as either “Insured,” “Uninsured,” “Medicaid,” “Prior to 2007,” or “Unknown.” Residence was originally divided into four categories (“Urban,” “Mostly Urban,” “Mostly Rural,” and “Rural”). For statistical modeling, we dichotomized the residence variable (urban or rural). Clinicopathologic factors such as primary tumor site, tumor grade, T‐ and N‐stage, and lymph node evaluation were also included.

### 2.5 Statistical analysis

Patient characteristics were summarized by socioeconomic status quintile using descriptive statistics including the median for continuous measures and proportions and frequencies for categorical measures. Kaplan–Meier and cumulative incidence function curves were plotted by SES along with accompanying logrank and Gray K‐sample tests. Models were fit for each of the two outcomes using backward selection with a 5% significance level. A Cox proportional hazard model was used for OS and a competing‐risk Fine and Gray regression model was used for CSS (Table 3). Hazard ratios (HR) with 95% confidence intervals (CI), and *p*‐values are presented. All analyses were performed using SAS 9.4.

## 3 RESULTS

The inclusion criteria identified 22,722 cases. Baseline characteristics of the cohort by SES quintile are shown in Table 1. A higher proportion of White patients (73.2%) comprised the highest SES quintile compared to the lowest (50%). However, a higher proportion of Black patients were in the lowest SES quintile (27.3%) compared to the highest quintile (4.5%). The lowest SES quintile comprised 39.6% of all Black patients though only 13% of all White patients. In the highest SES quintile, 69.6% of patients were insured, 3.4% had Medicaid, and 1.5% were uninsured. However, in the lowest SES quintile, 54.1% of patients were insured, 16.7% had Medicaid, and 5.4% were uninsured. The highest SES quintile had an increased proportion of patients with 12 or more lymph nodes identified (85.3%) compared to the lowest SES quintile (80.9%).

**TABLE 1:** Patient characteristics by socioeconomic status (SES)

	Lowest SES (0‐19th percentile)	Lower SES (20‐39th percentile)	Moderate SES (40‐59th percentile)	Higher SES (60‐79th percentile)	Highest SES (80‐99th percentile)
Number (%)	4667 (17.3)	5155 (19.2)	5493 (20.4)	5752 (21.4)	5828 (21.7)
Age (median)	62	63	63	63	63
Age					
>75 years	709 (15.2)	907 (17.6)	1000 (18.2)	1056 (18.4)	1093 (18.8)
≤75 years	3948 (84.8)	4248 (82.4)	4493 (81.8)	4696 (81.6)	4735 (81.2)
Sex					
Female	2385 (51.2)	2563 (49.7)	2696 (49.1)	2870 (49.9)	2844 (48.8)
Male	2272 (48.8)	2592 (50.3)	2797 (50.9)	2882 (50.1)	2984 (51.2)
Race/Ethnicity					
White	2324 (50.0)	3302 (64.1)	3765 (68.8)	4029 (70.2)	4247 (73.2)
Black	1271 (27.3)	685 (13.3)	524 (9.6)	417 (7.3)	259 (4.5)
Hispanic	797 (17.1)	781 (15.2)	657 (12)	547 (9.5)	364 (6.3)
Asian	222 (4.8)	353 (6.9)	502 (9.2)	728 (12.7)	916 (15.8)
American Indian Alaska Native	39 (0.8)	28 (0.5)	28 (0.5)	17 (0.3)	13 (0.2)
Insurance					
Insured	2517 (54.1)	3194 (62.0)	3613 (65.8)	3913 (68.0)	4055 (69.6)
Medicaid	779 (16.7)	473 (9.2)	397 (7.2)	329 (5.7)	197 (3.4)
Uninsured	250 (5.4)	193 (3.7)	153 (2.8)	117 (2.0)	88 (1.5)
Prior to 2007	1072 (23.0)	1256 (24.4)	1281 (23.3)	1341 (23.3)	1437 (24.7)
Unknown	39 (0.8)	39 (0.8)	49 (0.9)	52 (0.9)	51 (0.9)
Urban/Rural					
Rural	620 (13.3)	729 (14.1)	433 (7.9)	166 (2.9)	40 (0.7)
Mostly Rural	353 (7.6)	462 (9)	472 (8.6)	310 (5.4)	208 (3.6)
Mostly Urban	871 (18.7)	914 (17.7)	1100 (20)	1098 (19.1)	1239 (21.3)
Urban	2813 (60.4)	3050 (59.2)	3488 (63.5)	4178 (72.6)	4341 (74.5)
Primary Site					
Cecum	1072 (23.0)	1222 (23.7)	1240 (22.6)	1306 (22.7)	1328 (22.8)
Ascending Colon	819 (17.6)	902 (17.5)	1007 (18.3)	1032 (17.9)	1022 (17.5)
Hepatic Flexure	212 (4.6)	240 (4.7)	251 (4.6)	278 (4.8)	256 (4.4)
Transverse Colon	371 (8.0)	444 (8.6)	449 (8.2)	486 (8.5)	494 (8.5)
Splenic Flexure	199 (4.3)	194 (3.8)	178 (3.2)	216 (3.8)	205 (3.5)
Descending Colon	344 (7.4)	368 (7.1)	373 (6.8)	394 (6.9)	420 (7.2)
Sigmoid	1555 (33.4)	1693 (32.8)	1904 (34.7)	1952 (33.9)	2027 (34.8)
Large Intestine NOS	85 (1.8)	92 (1.8)	91 (1.7)	88 (1.5)	76 (1.3)
T Stage					
T1	198 (4.3)	226 (4.4)	270 (4.9)	311 (5.4)	375 (6.4)
T2	408 (8.8)	398 (7.7)	479 (8.7)	558 (9.7)	573 (9.8)
T3	3198 (68.7)	3596 (69.8)	3704 (67.4)	3856 (67)	3888 (66.7)
T4a	541 (11.6)	601 (11.7)	687 (12.5)	688 (12)	700 (12)
T4b	312 (6.7)	334 (6.5)	353 (6.4)	339 (5.9)	292 (5)
N Stage					
N1a	1501 (32.2)	1613 (31.3)	1746 (31.8)	1814 (31.5)	1880 (32.3)
N1b	1551 (33.3)	1707 (33.1)	1743 (31.7)	1904 (33.1)	1825 (31.3)
N2a	940 (20.2)	1015 (19.7)	1139 (20.7)	1145 (19.9)	1201 (20.6)
N2b	665 (14.3)	820 (15.9)	865 (15.8)	889 (15.5)	922 (15.8)
Lymph Node Evaluation					
12 or more	3765 (80.9)	4228 (82.0)	4635 (84.4)	4814 (83.7)	4969 (85.3)
<12 or unknown	892 (19.1)	927 (18)	858 (15.6)	938 (16.3)	859 (14.7)
Grade					
Well‐differentiated	265 (5.69)	269 (5.22)	256 (4.66)	237 (4.12)	297 (5.1)
Moderately‐differentiated	3317 (71.23)	3555 (68.96)	3807 (69.31)	3970 (69.02)	3893 (66.8)
Poorly/undifferentiated	1008 (21.64)	1276 (24.75)	1371 (24.96)	1474 (25.63)	1559 (26.75)
Unknown	67 (1.44)	55 (1.07)	59 (1.07)	71 (1.23)	79 (1.36)

### 3.1 Overall survival

**FIGURE 2:**
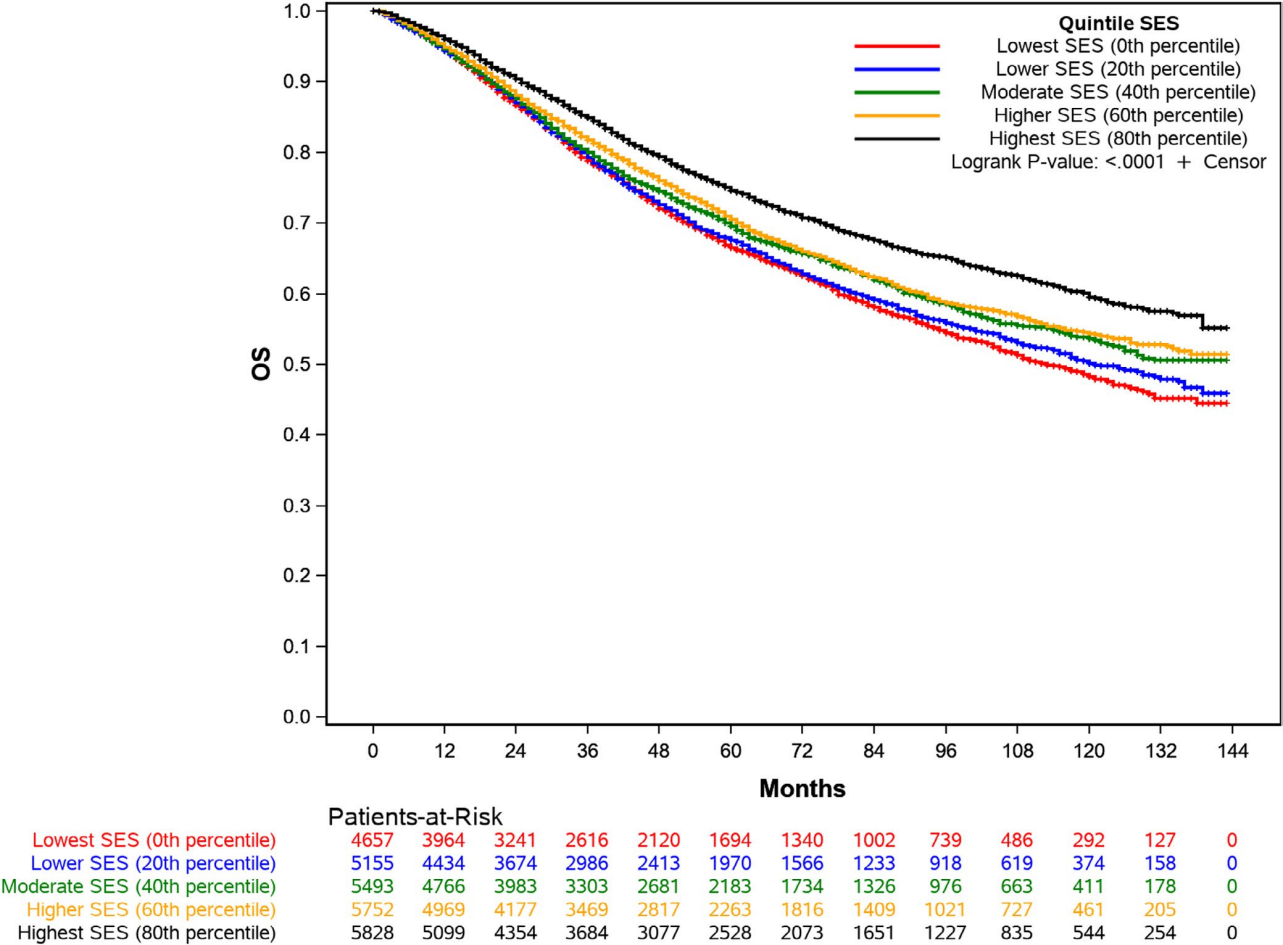
Kaplan‐Meier Curve of the Probability of Overall Survival

The median follow‐up for the cohort was 69 months and there were 8063 deaths. Median OS was 139 months (95% CI, 134‐NR) and the 5‐year OS rate was 70% (95% CI, 69–71%). There was a difference in OS by SES (*p* < 0.0001). Median overall survival was not reached for moderate, higher, and the highest SES quintiles; however, median OS was 121 and 113 months for the lower and lowest SES quintiles, respectively. The 5‐year OS rate was 66% for the lowest SES quintile and 75% for the highest SES quintile (Figure 2).

In adjusted analysis (Table 2), SES was associated with overall survival (*p* < 0.001). The hazard of death was increased for patients in all SES quintiles relative the highest SES quintile. The lowest SES quintile had the highest hazard of death (HR 1.33; 95% CI, 1.24–1.42) compared to the highest SES quintile. T4b tumors conferred the highest increase in hazard of death (HR 4.16; 95% CI, 3.52–4.91). Increasing age, male sex, Black race, higher T and N stage, grade, and tumor location were associated with an increased hazard of death.

**TABLE 2:** Relative hazard of death from colon adenocarcinoma: multivariable cox regression of overall survival

	Hazard ratio (95% confidence interval)	*p* value
Socioeconomic status (ref: highest SES)		
Lowest SES	1.33 (1.24–1.43)	<0.001
Lower SES	1.28 (1.20–1.38)	<0.001
Moderate SES	1.22 (1.14–1.31)	<0.001
Higher SES	1.19 (1.11–1.28)	<0.001
Age	1.03 (1.03–1.03)	<0.001
Male sex (ref: female)	1.23 (1.17–1.28)	<0.001
Race/Ethnicity (ref: White)		
Black	1.23 (1.14–1.31)	<0.001
Hispanic	1.03 (0.96–1.11)	0.430
Asian	0.84 (0.78–0.91)	<0.001
American Indian Alaska Native	0.94 (0.67–1.31)	0.710
Primary Site (ref: sigmoid)		
Cecum	1.19 (1.12–1.27)	<0.001
Ascending Colon	1.14 (1.06–1.22)	<0.001
Hepatic Flexure	1.22 (1.10–1.35)	<0.001
Transverse Colon	1.18 (1.08–1.28)	<0.001
Splenic Flexure	1.06 (0.93–1.20)	0.373
Descending Colon	1.12 (1.02–1.23)	0.020
Large Intestine NOS	1.25 (1.06–1.48)	0.008
T Stage (ref: T1)		
T2	1.23 (1.03–1.47)	0.023
T3	1.99 (1.71–2.32)	<0.001
T4a	3.00 (2.55–3.52)	<0.001
T4b	4.16 (3.52–4.91)	<0.001
N Stage (ref: N1a)		
N1b	1.23 (1.16–1.31)	<0.001
N2a	1.61 (1.50–1.71)	<0.001
N2b	2.42 (2.26–2.58)	<0.001
Grade (ref: Well‐differentiated)		
Moderately‐differentiated	1.03 (0.92–1.16)	0.566
Poorly/undifferentiated	1.25 (1.11–1.41)	<0.001
Unknown	1.26 (1.01–1.58)	0.044

Number of observations in the original data set = 27222. Number of observations used = 26815. Backward selection with an alpha level of removal of 0.05 was used. Urban/rural was removed from the model.

### 3.2 *Cancer*‐*specific survival*

**FIGURE 3:**
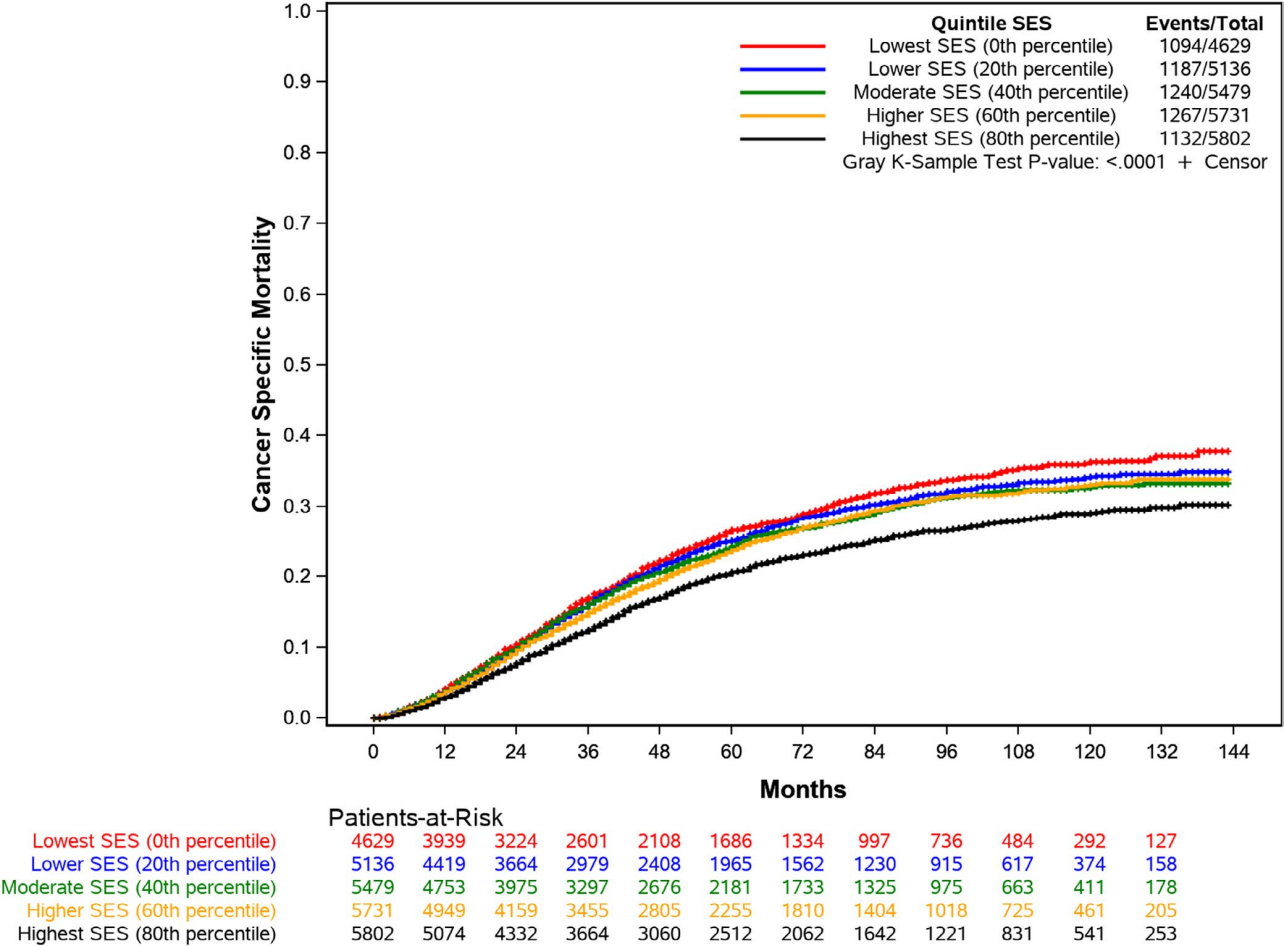
Cumulative Incidence of Cancer‐Specific Death by Socioeconomic Status

There were 5992 cancer‐specific deaths for a mortality rate of 22.1%. The median cancer‐specific survival was not reached for the cohort or SES quintiles (Table3). In adjusted analysis, SES remained associated with cancer‐specific survival (*p* < 0.001). Patients in the lowest SES quintile had an increased hazard of death (HR 1.23; 95% CI, 1.13–1.34) relative to the highest SES patients. Similar to overall survival, T4b tumors showed the highest relative odds of death (HR 5.93; 95% CI, 4.75–7.40). Relative to White patients, Black patients had increased hazard of cancer‐specific death (HR 1.29, 95% CI, 1.19–1.40) as did Hispanic patients (HR 1.11, 95% CI, 1.03–1.21) (Figure 3).

**TABLE 3:** Relative hazard of death from colon adenocarcinoma: multivariable fine and gray regression of cancer‐specific survival

	Hazard ratio (95% confidence interval)	Hazard ratio *p* value	Fine and Gray *p* value
Socioeconomic status (ref: highest SES)			<0.001
Lowest SES	1.23 (1.13–1.34)	<0.001	
Lower SES	1.17 (1.08–1.27)	<0.001	
Moderate SES	1.17 (1.07–1.27)	<0.001	
Higher SES	1.17 (1.08–1.27)	<0.001	
Age	1.02 (1.01–1.02)	<0.001	<0.001
Male sex (ref: female)	1.12 (1.06–1.18)	<0.001	<0.001
Race/Ethnicity (ref: White)			<0.001
Black	1.29 (1.19–1.40)	<0.001	
Hispanic	1.11 (1.03–1.21)	0.011	
Asian	0.90 (0.82–0.99)	0.026	
American Indian Alaska Native	0.95 (0.65–1.39)	0.800	
Primary Site (ref: sigmoid)			<0.001
Cecum	1.25 (1.17–1.35)	<0.001	
Ascending Colon	1.15 (1.06–1.24)	<0.001	
Hepatic Flexure	1.29 (1.14–1.46)	<0.001	
Transverse Colon	1.12 (1.01–1.24)	0.037	
Splenic Flexure	1.14 (0.99–1.31)	0.068	
Descending Colon	1.13 (1.02–1.26)	0.025	
Large Intestine NOS	1.22 (1.00–1.45)	0.052	
T Stage (ref: T1)			<0.001
T2	1.19 (0.93–1.51)	0.166	
T3	2.51 (2.04–3.08)	<0.001	
T4a	4.14 (3.34–5.13)	<0.001	
T4b	5.93 (4.75–7.40)	<0.001	
N Stage (ref: N1a)			<0.001
N1b	1.36 (1.26–1.46)	<0.001	
N2a	1.92 (1.78–2.08)	<0.001	
N2b	3.02 (2.79–3.27)	<0.001	
Grade (ref: Well‐differentiated)			<0.001
Moderately‐differentiated	1.06 (0.93–1.22)	0.380	
Poorly/undifferentiated	1.41 (1.10–1.82)	0.007	
Unknown	1.33 (1.15–1.53)	<0.001	

## 4 DISCUSSION

In this study, SES was associated with disparities in OS and CSS for stage III colon cancer patients treated with surgery and adjuvant chemotherapy, the standard of care. The lowest SES patients had a 33% increase in overall mortality and a 23% increase in cancer‐specific mortality relative to the highest SES patients. Additionally, racial disparities in CSS were observed, with Black and Hispanic patients experiencing higher cancer‐specific mortality. Collectively, these findings show that despite guideline‐based treatment, survival disparities by SES and race persist for stage III colon cancer patients and suggest that post‐treatment paths, notably follow‐up and surveillance as well as treatment quality after recurrence, may contribute to this disparity.

Several randomized trials have shown a survival benefit when adjuvant chemotherapy is given.^6^ Despite these benefits, recurrence remains frequent; roughly 30%–40% of patients will eventually experience a recurrence and the majority will occur within 2 years of surgery.^19^, ^20^, ^21^ Traditionally, recurrence was associated with a poor prognosis; however, recent evidence suggests that select patients with locoregional and metastatic disease can be resected with improved long‐term outcomes.^15^, ^22^ While the impact on OS remains unclear, some randomized clinical trials have shown that intensive follow‐up may lead to earlier detection of recurrence and subsequent surgical resection.^23^, ^24^, ^25^ In the FACS trial, more intensive follow‐up led to over three times increased odds of curative‐intent surgery compared to minimal, symptom‐driven follow‐up. Furthermore, in the CEAwatch trial, the cohort under surveillance had improved survival compared patient who experienced recurrence that was detected by self‐report.^26^ Nevertheless, other studies, including randomized trials, have found that greater intensity of surveillance is not associated with improved outcomes.^27^, ^28^

While the exact intensity of follow‐up surveillance remains uncertain, these results have limited generalizability given the low rate of surveillance in the United States.^28^ Data show that 75% of post‐surgical patients do not receive the minimum recommended surveillance and there has been minimal improvement over time.^16^ A more recent retrospective cohort study showed that 23% of patients who underwent curative‐intent at several National Cancer Institute‐designated Comprehensive Cancer.

Centers received guideline concordant surveillance and nearly half of stage III patients did not undergo CT imaging within 14 months of surgery.^29^ Although specific risk factors for failure to surveil stage III colon cancer patients remain unknown, social determinants, including socioeconomic and insurance status, and the cumulative financial toxicity of cancer treatment, may contribute to the low rate of follow‐up.

Several studies have shown that lower SES leads to less colon cancer treatment and worse survival.^30^, ^31^, ^32^, ^33^ However, these cohorts have typically included patients of all or non‐metastatic stages or focused on disparities in receipt of surgery.^34^ Abdel‐Rahman used the SEER census‐tract dataset to evaluate a cohort of stage I‐III colon cancer patients who underwent surgery and found that SES was associated with cancer‐specific survival although adjuvant chemotherapy was not included in the analysis.^35^ This study addressed a gap in knowledge by assessing the impact of SES on survival for stage III patients who receive standard of care therapy.

As stage III patients are at the highest risk of recurrence and subsequent cancer‐specific death, there are several implications of these findings. As the impact of financial toxicity on cancer patients becomes clearer, there is a need to clarify the mechanisms by which this process impacts colon cancer patients. Data suggest that low income, under and uninsured, and younger patients are the most effected by financial toxicity, which can lead to treatment and/or surveillance non‐adherence.^36^, ^37^ Furthermore, survey data showed that nearly half of patients with stage III colon cancer did not retain their job due to their cancer diagnosis and treatment, which also may influence clinical follow‐up and treatment.^38^ Another possible explanation is that the highest SES patients benefit from referral for potential resection in patients with recurrence. Data suggest that potentially curative surgical therapies are underutilized for metastatic colon cancer patients and surgical referral is uncommon.^39^, ^40^

Race has also been shown to be a predictor of colon cancer survival; however, randomized trials have not demonstrated racial disparities for Black patients receiving adjuvant therapy for colon cancer relative to white patients.^7^ However, the data presented here demonstrate that after adjustment with a granular measure of SES, Black and Hispanic patients had an increased relative risk of colon cancer death compared to White patients. Prior studies have suggested a substantial correlation between receipt of adjuvant chemotherapy for colon cancer and poverty, inadequate insurance coverage, and African American race.^41^ Taken together, this evidence suggests that structural racism, broadly viewed as the social (and economic) forces and arrangements that create disproportionate harm and contribute to worse health outcomes for Black patients, exerts an influence in this cohort of patients.^42^, ^43^, ^44^ Although assessing the interaction of SES, race, and insurance was infeasible due to low numbers of deaths within interaction strata, Black patients comprised only 4.5% of the highest SES cohort despite making up 11.8% of total patients. Prior studies have shown that Black patients are also more likely to receive no surveillance testing for stage II and III colon cancer, suggesting a potential avenue to address this disparity.^45^

There are several limitations to consider when interpreting the results of this analysis. The SEER census tract database does not contain data regarding whether patients received guideline‐based pre‐ or post‐operative staging, which may impact stage accuracy and prognosis. Additionally, SEER does not provide data regarding follow‐up or recurrence. Furthermore, data on comorbidities are not collected by SEER registries, which may serve as a potential confounder. However, by presenting CSS in addition to OS, competing causes of death, such as those due to comorbidities, were censored. Finally, the dataset also does not provide data regarding adjuvant chemotherapy regimen and duration, which may also impact the results.

In conclusion, stage III colon cancer patients in the highest SES had improved overall and cancer‐specific survival after adjustment for potential demographic and clinical confounders. The specific causes of this disparity are unknown and unable to be identified in this exploratory analysis. However, adherence to surveillance regimens and early detection of recurrence may drive survival outcomes and high SES may serve as a buffer against financial toxicity and improve access to high‐quality care and referrals. Future efforts should aim to identify the specific social‐determined risk factors associated with these downstream disparities.

## Conflict of interest

The authors declare no conflicts of interest.

## AUTHOR CONTRIBUTIONS

Study conceptualization/design: AD, JK, MN, NCB, EC, and BDP; Data curation: AD, BDP, MN, and NCB; Formal analysis/investigation: MN and NCB; Manuscript writing: AD, JK, and BDP; Manuscript review/approval: AD, JK, MN, NCB, SON, II, SIF, SPD, JS, SD, EC, and BDP; This manuscript was not an invited submission. Presented at the 2020 American Society of Clinical Oncology (ASCO) Annual Meeting, May 29–31, 2020.

## Supporting information

Table S1
