# Supplementary material for: The impact of socioeconomic status on survival in stage III colon cancer patients: A retrospective cohort study using the SEER census‐tract dataset
Source: Cancer Med. 2021 Jun 30;10(16):5643–52. doi: 10.1002/cam4.4099 (PMC8366079; doi:10.1002/cam4.4099)
Supplement: Supplementary file 1 — Table S1 [file CAM4-10-5643-s001.docx]

**Supplemental Table 1: Selection Criteria for Colon Primary Site (A) and Selection Criteria for Adenocarcinoma Histology (B).**

**A.**

C18.0-Cecum,

C18.2-Ascending colon,

C18.3-Hepatic flexure,

C18.4-Transverse colon,

C18.5-Splenic flexure of colon,

C18.6-Descending colon,

C18.7-Sigmoid colon,

C18.8-Overlapping lesion of colon and

C18.9-Colon, NOS.

**B.**

8140/3: Adenocarcinoma, NOS

8141/3: Scirrhous adenocarcinoma

8144/3: Adenocarcinoma, intestinal type

8210/3: Adenocarcinoma in adenomatous polyp

8211/3: Tubular adenocarcinoma

8220/3: Adenocarcinoma in adenomatous polyposis coli

8221/3: Adenocarcinoma in multiple adenomatous polyps

8255/3: Adenocarcinoma with mixed subtypes

8260/3: Papillary adenocarcinoma, NOS

8261/3: Adenocarcinoma in villous adenoma

8262/3: Villous adenocarcinoma

8263/3: Adenocarcinoma in tubulovillous adenoma.
